# Capturing deep partial-thickness cartilage loss with semiquantitative scoring: A supplemented MRI Osteoarthritis Knee Score (sMOAKS)

**DOI:** 10.1016/j.ostima.2025.100272

**Published:** 2025-06-07

**Authors:** Faysal Altahawi, Richard Lartey, Nancy Obuchowski, Xiaojuan Li, Carl S. Winalski

**Affiliations:** a Program of Advanced Musculoskeletal Imaging (PAMI), Cleveland Clinic, Cleveland, OH, USA; b Department of Diagnostic Radiology, Imaging Institute, Cleveland Clinic, Cleveland, OH, USA; c Department of Biomedical Engineering, Lerner Research Institute, Cleveland Clinic, Cleveland, OH, USA; d Department of Quantitative Health Sciences, Lerner Research Institute, Cleveland Clinic, Cleveland, OH, USA

**Keywords:** Semi-quantitative score, Reliability, MOAKS, sMOAKS, Knee MRI, Osteoarthritis, Cartilage score, Post-traumatic osteoarthritis

## Abstract

**Objective:**

We propose a supplement to MOAKS (MRI Osteoarthritis Knee Score) for capturing >50 % partial thickness cartilage loss on knee MRI and measure reader agreement.

**Design:**

MOAKS scores 2 severity levels of cartilage damage (any loss, full-thickness loss) within knee subregions with lesional area graded 0–3. We propose supplemented MOAKS (sMOAKS) by adding a similarly graded third level assessment for deep cartilage loss (DCL), >50 % thickness, in addition to traditional MOAKS for improved granularity of partial thickness cartilage loss. Using sMOAKS, two radiologists independently graded cartilage subscores for 40 knees and rescored 20 knees. Consolidated inter-reader and intra-reader agreement was calculated with kappa values for the DCL level supplement. To measure agreement for one example of a more granular combined sMOAKS outcome, coverage probability reader agreement was calculated for a scaled cartilage damage score (CDS), a summed normalized score (0–100) with equal weighting to articular surfaces combining subregion scores.

**Results:**

DCL represented 27.8 % (63/227) of partial but not full-thickness cartilage loss subregion interpretations. Pooled subregion DCL involving >10 % surface area demonstrated 97.7 % (*ĸ* = 0.71) inter-reader agreement and 98.8 % (*ĸ* = 0.78) intra-reader agreement. For greatest subregion DCL size, weighted ĸ agreement was 0.73/0.82 (inter-reader/intra-reader) for articular surfaces and 0.75/0.83 for joint compartments. At 90 % CDS intra-reader agreement coverage, inter-reader CDS agreement values were 83 %, 82 %, and 78 % for surfaces, compartments, and whole joints, respectively.

**Conclusions:**

There is substantial agreement for deep cartilage loss detection using sMOAKS across varied analysis methods. Further assessment will determine when the added granularity of sMOAKS is beneficial.

## Introduction

Knee osteoarthritis (OA) presents a substantial human and economic burden in society [1,2]. Articular cartilage damage and loss are considered a hallmark of OA, whether the OA occurs as the result of trauma or from other causes. Morphological assessment of articular cartilage has therefore been considered instrumental in characterizing and assessing the progression of OA. The direct grading of knee articular cartilage has evolved from the use of arthroscopy [3,4] to the use of noninvasive magnetic resonance imaging (MRI) methods [[5], [6], [7]].

With the goal of achieving a useful measurement for assessing OA prevention and treatment strategies in clinical trials, a number of semiquantitative MRI grading systems for evaluating knee OA progression have been proposed, including the Whole-Organ MRI Score (WORMS), the Knee Osteoarthritis Scoring System (KOSS), the Boston-Leeds Osteoarthritis Knee Score (BLOKS), and the MRI Osteoarthritis Knee Score (MOAKS) [[8], [9], [10], [11]]. These semiquantitative measures were developed mainly using databases containing images from middle-aged patients, largely those with established OA [[8], [9], [10], [11]].

In these MRI grading systems, articular cartilage damage subscores are based on the depth of any cartilage defects, and articular damage is thus recorded across 2 potential levels of cartilage loss: partial-thickness or full-thickness [[8], [9], [10], [11]]. For partial-thickness cartilage defects, there is typically no differentiation in these systems between shallow (i.e., ≤50 % loss of normal cartilage thickness) and deep (>50 % thickness loss, but not full thickness) defects. However, surgical management of partial thickness cartilage damage often depends on the depth of cartilage loss, whereby deep partial thickness cartilage defects are considered for surgical management the same as full thickness defects [12]. Additionally, with the addition of an intermediate partial-thickness cartilage score for deep (>50 % thickness loss) defects, the granularity of these systems for assessing early OA development and progression is increased, which may be important for young patient cohorts that often have post-traumatic OA [5,13].

Outcome analyses of knee MRI semi-quantitative cartilage loss scores are varied in cohort and longitudinal studies, and can include counts of a thresholded severity of cartilage defects, measurement of longitudinal changes in subregions, maximum joint or regional scores, summation of regional scores, or a combination therein [14]. Increasing the granularity of cartilage subscores through the differentiation of shallow and deep cartilage loss may increase the sensitivity of the scoring instrument for clinically meaningful deep or full thickness cartilage loss in thresholded analyses, longitudinal change in partial thickness cartilage defects, and better differentiation in overall cartilage damage for summed scores. Tools currently used to improve granularity, such as the capture of “within grade changes” in longitudinal studies, could still be applied to such a system to provide even finer detail [15]. In the case of summed scores for cartilage damage, the methods vary across the literature. Since frequent lack of normalization and standardization across used summed score methods can make the overall impact of lesions vary by location and create the possibility to “drown out” severity of focal cartilage damage, summed scores are less commonly used [14,16].

To this end, we defined a supplementation of the MOAKS cartilage subscore with the addition of a third level cartilage loss score for deep, >50 % thickness, partial-thickness loss for each subregion. We then measured the reader reliability of the proposed supplemented MOAKS (sMOAKS) cartilage assessment in a patient population at risk for post-traumatic OA among observers and for different analyses methods, including size thresholded subregional scores (to capture lesions that may be considered for cartilage repair), maximum regional or whole joint scores, as well as summed scores with normalized cartilage damage scores (CDSs) scaled to 100 to evaluate cartilage loss in more consolidated regions, i.e. articular surfaces, joint compartments, and the whole joint [14,17,18].

## Methods

### sMOAKS cartilage subscore

For each cartilage subregion, the traditional MOAKS cartilage subscore provides size grades based on the involved surface area relative to each subregion (0: none, 1: <10 %, 2: 10 %−75 %, 3: >75 %) at two depth levels of cartilage damage: 1. “any cartilage loss (including any partial- or full-thickness loss)” and 2. “full-thickness cartilage loss” [8]. Conventional notation for MOAKS cartilage lesions utilizes the size grading of any cartilage depth, followed by a period, followed by the size grading of full-thickness cartilage depth (ie 1.0, 2.1, etc.). The proposed sMOAKS subscore adds an additional depth level for >50 % thickness deep cartilage loss (DCL), consisting of any cartilage loss more than half of the normal cartilage thickness (including full-thickness loss) using the same size grading, for a total of 3 depth levels of cartilage damage, each similarly graded 0 – 3 for the involved surface area relative to each subregion (Table 1). Notation for sMOAKS cartilage lesions utilizes the size grading of any cartilage depth, followed by a period, followed by the size grading of DCL, followed by a period, followed by the size grading of full-thickness cartilage depth (ie 2.1.1, 2.2.0, etc.).

**Table 1:** Size grades definition for cartilage subscores in sMOAKS (supplemented MOAKS) and traditional MOAKS.

Feature	Any cartilage loss (partial- or full-thickness):	Any deep cartilage loss (partial-thickness >50 % and full-thickness):	Full-thickness cartilage loss
**Scoring System**	**sMOAKS** **Traditional MOAKS**	**sMOAKS only**	**sMOAKS** **Traditional MOAKS**
**Grade**	Grade 0: none	Grade 0: none	Grade 0: none
	Grade 1: <10 % subregion cartilage surface	Grade 1: <10 % subregion cartilage surface	Grade 1: <10 % subregion cartilage surface
	Grade 2: 10 %−75 % subregion cartilage surface	Grade 2: 10 %−75 % subregion cartilage surface	Grade 2: 10 %−75 % subregion cartilage surface
	Grade 3: >75 % subregion cartilage surface	Grade 3: >75 % subregion cartilage surface	Grade 3: >75 % subregion cartilage surface

MOAKS, Magnetic Resonance Imaging Osteoarthritis Knee Score.

### Study design

This study was approved by the Institutional Review Board and was performed in compliance with the Helsinki Declaration and signed informed consent obtained from all subjects. A subcohort of 219 participants from the Multicenter Orthopaedic Outcomes Network (MOON) prospective nested cohort [16], all of whom underwent unilateral anterior cruciate ligament (ACL) reconstruction between 2005 and 2012, were recruited from three sites for 10-year follow-up (recruitment ongoing). The MOON cohort was selected due to the established propensity to develop premature post-traumatic OA, serving as an excellent “early OA” model, when differentiation between deep and shallow partial thickness cartilage loss may be most valuable [16]. The inclusion criteria involved enrollment in MOON Onsite Study, 12–33 years old at baseline (22–45 years for this study), ACL tear during a sport, no previous knee injury, no graft rupture during follow-up, no history of surgery on the contralateral knee, and no MRI contraindications. Exclusion criteria include history of surgery on contralateral knee or a fear of enclosed spaces.

Bilateral knee MR images were obtained (i.e., ACL reconstruction and nonoperated knees) using 3T MR systems (Siemens Skyra Fit, Erlangen, Germany; or Philips Ingenia, Best, Netherlands) with transmit/receive knee coils (1Tx/15Rx or 1Tx/16Rx). The imaging protocol was harmonized among sites and a rigorous quality control process applied [17]. For this study, sagittal, coronal, and axial 2D turbo spin-echo (TSE), sagittal 3D dual-echo steady-state (DESS), and sagittal 3D TSE (SPACE or VISTA) images with coronal and axial multiplanar reconstructions were used for review (Table 2). Additionally obtained sagittal MAPSS T1rho/T2 and T2 MESE acquisitions were available but were not reviewed (not shown in Table 2), and no relaxation parameter maps were available to the readers.

**Table 2:** Magnetic resonance imaging protocol parameters.

Characteristic	Sequence				
	Sagittal proton density fat-saturated TSE	Coronal proton density fat-saturated TSE	Axial proton density fat-saturated TSE	Sagittal 3D TSE (SPACE/VISTA)	Sagittal DESS
Acquisition time, min:s	2:18	1:22	1:31	4:28	5:56
Repetition time, ms	2490	2430	6910	1000	17.55
Echo time, ms	18	18	20	28	6.02
Matrix, frequency × phase	320 × 256	320 × 256	256 × 256	320 × 304	384 × 307
Number of slices	33	37	41	160	160
Field of view, mm	140	140	140	140	140
Slice thickness, mm	3	3	3	0.7	0.7
Skip, mm	0.3	0.3	0.3	0	0
Flip angle	150°	150°	150°	Variable	25°
Bandwidth, Hz/pixel	300	300	300	422	186
Fat suppression	Fat saturated	Fat saturated	Fat saturated	No	Fat saturated

TSE, turbo spin-echo; SPACE, sampling perfection with application-optimized contrasts; VISTA, volume isotropic turbo spin-echo acquisition; DESS, dual-echo steady-state.

A total of 40 knee MR images (30 injured ACL reconstructed knees and 10 uninjured knees) were selected randomly from the available knees for review. MR images were independently scored by two fellowship-trained musculoskeletal radiologists with 5 and 34 years of subspecialty experience using the sMOAKS cartilage subscore method. Each subregion was graded with scores ranging from 0 to 3 for any cartilage loss, any DCL, and any full-thickness cartilage loss. After a washout period of at least 1 month, rescoring of 20 of the knees was performed by each radiologist independently and without knowledge of the initial scores.

**Fig. 1:**
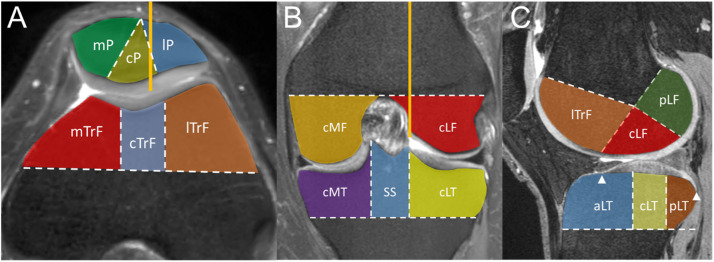
Subregion definitions*. Axial (A), coronal (B), and sagittal (C) representations of the subregions used in this study. Different from the traditional MOAKS, patellar and trochlear surfaces were divided into 3 subregions each. The subregion divisions were based on the subchondral bone plate margins (marked by arrowheads in C). For comparison, the orange lines in A and B show the divisions for traditional MOAKS where the patellar apex is included in the medial patella and the lateral wall of the intercondylar notch divides medial from lateral femur. mP = medial patella; cP = central patella; lP = lateral patella; mTrF = medial trochlea; cTrF = central trochlea; lTrF = lateral trochlea; cMF = central medial femoral condyle; cLF = central lateral femoral condyle; cMT = central medial trochlea; cMT = central medial tibial plateau; SS = supspinous region; cLT = central lateral tibial plateau; aLT = anterior lateral tibial plateau; pLT = posterior lateral tibial plateau. *Definitions are detailed by Xie, et al. [17] and summarized in the Supplemental Material.

For this study, the cartilage surfaces were divided into 16 subregions as described by Xie et al. [17] (Fig. 1, Supplementary Material). This division differs from traditional MOAKS in that the patella and trochlea were divided into three subregions each, as proposed by Eckstein, et al. [19] instead of two (Fig. 1), since many cartilage defects are centered at the patellar apex and central trochlea groove. Additionally, margins of all subregions were defined by the subchondral bone plate, and bony regions not expected to have overlying articular cartilage were excluded. The femoral condyle division was based on the projection of a line tangential to the roof of the intercondylar notch (Blumensaat’s line) that separates the trochlea from the femoral condyles. An orthogonal line bisecting this line at the center of each condyle separated the posterior and central femoral condyles. The patellar and trochlear divisions were defined by identifying the articular central apex and ridge points, with the central subregion defined as one-third of the distance from the central point to the medial and lateral margins in the axial plane. While these formalized subregion division lines could be drawn using the imaging visualization system tools, for this study the readers visually estimated the subregions borders based on these definitions.

### sMOAKS CDS (cartilage damage score)

To test agreement of a more granular outcome measure calculated from sMOAKS scores, a method for summing and consolidating subregion scores into a scaled CDS for articular surfaces, joint compartments, and the entire joint was developed and reader agreement measured. A summed, ordinal score was calculated for each subregion for a possible score ranging from 0 (no cartilage loss) to 9 (full-thickness cartilage loss involving >75 % of the subregion surface area). Each total subregion cartilage summed value (range, 0–9) was divided by 9 and then multiplied by 100 to provide a 100-point scaled subregion CDS. To calculate CDSs for the six articular surfaces (medial femoral condyle, medial tibial plateau, lateral femoral condyle, lateral tibial plateau, patella, and trochlea), the sum of the appropriate subregions’ scaled CDS was divided by the number of consolidated subregions (two or three) for a 100-point “articular surface CDS.” The scaled CDSs for the three joint compartments (medial, lateral, and patellofemoral) and the whole joint were calculated by averaging the corresponding articular surface CDSs so that each of the incorporated articular surfaces contributed equally to the compartment or whole joint CDS. Thus, a CDS of 0 indicates no cartilage loss while a CDS of 100 reflects full-thickness cartilage loss involving >75 % of the surface area of every included subregion.

### Statistical analysis

**sMOAKS DCL grading.** Cohen’s kappa statistics were calculated for pooled subregion intra-reader and inter-reader agreement for the presence of at least grade size 2 DCL (>50 % cartilage thickness loss [including full-thickness loss] involving at least 10 % of the subregion surface area). This was felt to correspond to the lowest equivalent score that may be considered for surgical cartilage repair [12]. Inter-reader and intra-reader linearly weighted kappa agreement for size grading of 0 to 3 for DCL was based on ordinal binning for each subregion and was calculated at the pooled subregion level. Because damage severity is often considered to be the worst level of cartilage loss, similarly weighted kappa agreement was assessed for the highest subregion DCL size grading at the pooled surface and compartment levels. Additionally, 95 % confidence intervals (CIs) were corrected to account for the clustered nature of the data through the use of logistic regression models with generalized estimating equations for binary outcomes and bootstrapping at the subject level for kappa.

**sMOAKS CDS.** Coverage probability plots were constructed for the semicontinuous composite CDS, which is representative of the composite subregion sMOAKS cartilage loss within a surface, compartment, or the whole joint and scaled to 100 [16,20]. Intra-reader and inter-reader CDS agreement curves were constructed for the pooled surface, compartment, and whole joint CDS. These curves demonstrated the proportion of interpretations that agreed based on variable definitions of agreement, such that the y-intercept represented the proportion of cases with exact CDS agreement. Inter-reader agreement was evaluated at each level at the threshold for 90 % intra-reader coverage (i.e. the CDS difference corresponding to 90 % intra-reader agreement).

## Results

Forty knee MRs from 32 total patients (8 had both knees included in the analysis) were reviewed. At the time of imaging, mean patient age was 37 years (range 29–51 years), mean body mass index (BMI) 27.5 (range 19–55), with 17 males (53 %). Of the 40 knee MR images reviewed, 30 had undergone ACL reconstruction (10 nonoperative knees) and 24 were right knees (16 left knees).

### sMOAKS subregion score

Across all subregion findings, there were 227 subregion interpretations of partial-thickness cartilage loss of any depth (i.e. non-full thickness cartilage defects) representing 11.8 % (227/1920) of all subregion interpretations. Of those, there was DCL (>50 % thickness) of any size in 63 (27.8 %, 63/227). Of those interpretations with DCL but not full-thickness loss, 25.4 % (16/63) involved >10 % of the surface area (i.e. size grades 2 or 3).

Pooled subregion agreement for grade 2 or 3 sized (i.e. >10 % involved surface area) subregion DCL was 97.7 % (625/640) for inter-reader agreement (*ĸ* = 0.71, 95 % CI: 0.50–0.82) and 98.8 % (632/640) for intra-reader agreement (*ĸ* = 0.78, 95 % CI: 0.66–0.94) (Table 3, Table 4). Inter-reader percent agreement for individual subregion scores ranged from 92.5 % to 100 %. Inter-reader agreement across all 0 to 3 size grades for subregion DCL was 93.9 % (601/640) [95 % CI: 91.8 %−96.5 %]. Agreement for all subregion DCL size grades pooled for all subregions yielded linear-weighted ĸ agreement values of 0.43 (95 % CI: 0.28–0.54) for inter-reader agreement and 0.50 (95 % CI: 0.40–0.60) for intra-reader agreement.

**Table 3:** Inter-reader agreement for subregion DCL (>50 % thickness) grading by areal size, pooled.

Reader 2	Reader 1			
	No DCL	<10 % DCL	10 %−75 % DCL	>75 % DCL
No DCL	572	13	2	0
<10 % DCL	10	11	3	1
10 %−75 % DCL	5	4	15	1
>75 % DCL	0	0	0	3

DCL, deep cartilage loss.

**Table 4:** Agreement for subregion >50 % thickness deep cartilage loss (DCL), pooled.

Agreement metric	Inter-reader	Intra-reader
Exact agreement for subregion presence/absence of >10 % surface area DCL (n/N)	97.7 % (625/640)	98.8 % (632/640)
Kappa for subregion presence/absence of >10 % surface area DCL [95 % CI*]	0.71 [0.50–0.82]	0.78 [0.67–0.94]
Weighted kappa for all sizes of subregion DCL (size grades 0 to 3) [95 % CI*]	0.43 [0.28–0.54]	0.50 [0.40–0.60]
Weighted kappa for maximum subregion DCL size grade within an articular surface [95 % CI*]	0.73 [0.58–0.80]	0.82 [0.73–0.86]
Weighted kappa for maximum subregion DCL size grade within a joint compartment [95 % CI*]	0.75 [0.59–0.85]	0.83 [0.73–0.88]

*Bootstrap 95 % confidence interval (CI), with resampling at the subject level.DCL, deep cartilage loss.

n/*N* = number of agreements/number of subregions scored (640 = 40 knees x 16 subregions).

Analysis of the highest subregion DCL graded size within articular surfaces yielded pooled weighted ĸ agreement values of 0.73 (95 % CI: 0.58–0.80) for inter-reader agreement and 0.82 (95 % CI: 0.73–0.86) for intra-reader agreement (Table 4). Agreement for individual surfaces ranged from weighted ĸ 0.52 to 0.86 for inter-reader agreement and 0.72 to 1.0 for intra-reader agreement (Supplementary Table 1). Analysis of the highest subregion DCL graded size within joint compartments yielded pooled weighted ĸ agreement values of 0.75 (95 % CI: 0.59–0.85) for inter-reader agreement and 0.83 (95 % CI: 0.73–0.88) for intra-reader agreement. Individual compartment weighted ĸ agreement values ranged from 0.67 to 0.86 for inter-reader agreement and 0.74 to 0.88 for intra-reader agreement (Supplementary Table 2).

### sMOAKS CDS

**Fig. 2:**
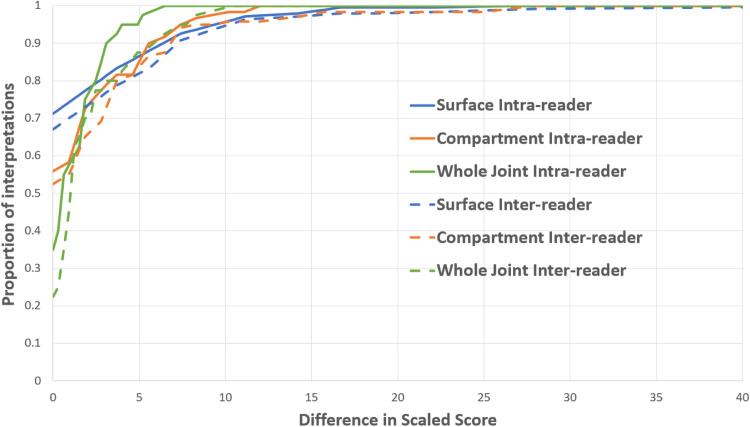
Coverage probability plots for differences in the sMOAKS cartilage damage score (CDS). CDS, a semicontinuous composite cartilage loss score scaled to 100, at the surface level (6 articular surfaces are pooled, *N* = 240 total interpretations), compartment level (3 compartments are pooled, *N* = 120 total interpretations), and whole joint level (*N* = 40 total interpretations). The y-intercept indicates the proportion of cases for which there was exact CDS agreement.

**Fig. 3:**
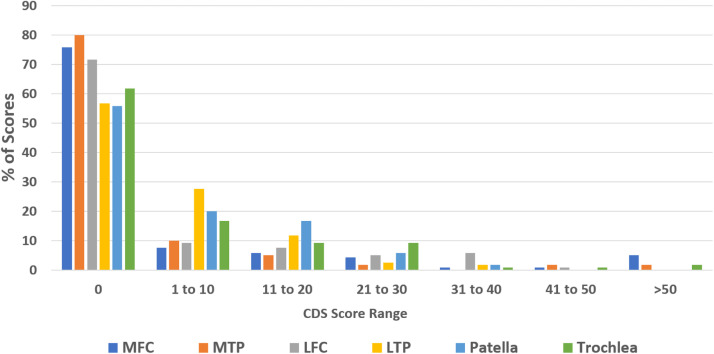
Distribution of sMOAKS cartilage damage score (CDS) by articular Surface level sMOAKS CDS is the sum of subregion sMOAKS cartilage scores (0 to 9 in each subregion) within each surface, rescaled to 100. MFC = medial femoral condyle; MTP = medial tibial plateau; LFC = lateral femoral condyle; LTP = lateral tibial plateau. Results are based on all interpretations (*n* = 240).

Pooled subregion inter-reader agreement for overall exact summed cartilage loss grading (0–9) was 83.0 % (531/640) [95 % CI: 77.5 %−89.1 %]. For scaled CDS (range 0 – 100) at the articular surface, compartment, and whole joint levels, 90 % intra-reader coverage yielded thresholds for agreement of score differences of 6 or less at the surface level, 5 or less at the compartment level, and 3 or less at the whole joint level (Fig. 2). Pooled inter-reader agreement values for the CDS at these thresholds were 83.3 % (200/240) [95 % CI: 78.0 %−88.7 %], 81.7 % (98/120) [95 % CI: 73.4 %−90.1 %], and 77.5 % (31/40) [95 % CI: 67.7 %−94.8 %] at the surface, compartment, and whole joint levels, respectively. The distribution of scaled cartilage loss CDS scores at the articular surface level for this cohort is shown in Fig. 3.

## Discussion

In this study, we supplemented the MOAKS articular cartilage assessment with an additional measure for deep partial-thickness (>50 % thickness) cartilage loss (DCL) and measured the reliability of this score. With this sMOAKS method, both the standard MOAKS cartilage score and the deep partial-thickness score are simultaneously captured, since the original 2 severity levels of cartilage damage are also recorded. For sMOAKS, we found substantial agreement between two musculoskeletal radiologists for the detection of DCL involving >10 % of the subregion surface area as well as for grading the maximum subregion DCL size for each of the six articular surfaces. Our measured agreement was similar to that published for traditional MOAKS cartilage grading [8].

**Fig. 4:**
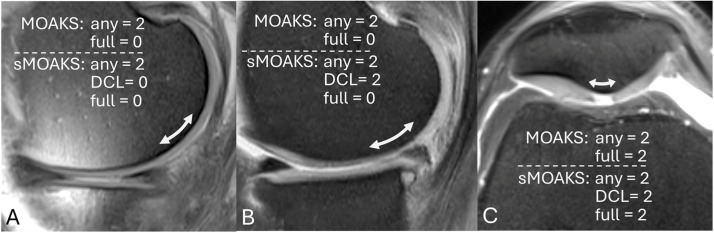
Comparison of sMOAKS with traditional MOAKS for lesion depth. Proton Density fat saturated images from three patients with grade 2 size cartilage loss (curved arrows). The 2 depth level assessment (any cartilage loss & full thickness defect) of MOAKS assigns the same grades regions of shallow (A) and deep (B) partial-thickness cartilage loss and only differentiates the full-thickness defect, as seen in the central patellar subregion in (C); MOAKS would capture (A) as 2.0, (B) as 2.0, and (C) as 2.2. With 3 depth level assessment, sMOAKS differentiates all 3 types of defects by recording that the lesion in (B) has deep cartilage loss (DCL); sMOAKS would capture (A) as 2.0.0, (B) as 2.2.0, and (C) as 2.2.2. cP = central patella, lP = lateral patella, mP = medial patella.

Since partial-thickness cartilage loss is a common phenomenon, particularly in the early development of OA, separation of deep and superficial cartilage damage by MRI scoring systems may be beneficial for certain patient cohorts. Before the development of established OA, it can be difficult to tease out meaningful cartilage damage burden and disease progression without separation of partial-thickness cartilage defects into shallow and deep cartilage loss. Two knees with similarly sized and located partial-thickness cartilage loss can have a clear difference in the severity based on the depth of partial-cartilage loss; these differences may not be captured using traditional MRI grading systems but would be captured using sMOAKS (Fig. 4).

In addition, shallow and deep partial-thickness cartilage defects may be treated differently. For example, most surgeons do not consider cartilage repair surgery appropriate for shallow cartilage defects, but will consider repair for deep defects, especially when symptomatic [12,21,22]. Therefore, sMOAKS may prove useful for isolating cases of shallow cartilage loss for which the clinical impact may not be as pertinent. Within our small cohort of relatively young patients at risk for post-traumatic OA, we found that 11.8 % of subregion gradings were partial-thickness defects, and sMOAKS was able to identify deep, partial-thickness defects in 27.8 % of these cases. This differentiation between deep and shallow partial-thickness, not previously captured in many knee MRI grading systems, may prove to be a clinically relevant transition in the progression of OA. The ability to detect changes in cartilage loss severity can be critical in identifying early OA disease progression, particularly in longitudinal research studies using knee MRI to evaluate the clinical impact of early OA or to assess therapies [23,24].

Outcome measures based on the sMOAKs approach may be analyzed by any of the same counting, thresholding, summation and change/delta methods with MOAKS since the additional category for deep cartilage loss assessment is captured in a similar method. Additionally, within-grade change may also be considered, as previously proposed for WORMS [15]. The increased granularity of cartilage assessment offered by sMOAKS lends itself not only to identification of more clinically meaningful deep partial thickness defects, but also to creating a more continuous outcome variable that may prove valuable for cohort studies and clinical trials as an additional statistical metric for quantification of knee OA [16]. Such an approach may help with the comparison of morphological cartilage assessments and the increasingly used continuous quantitative variables measured by compositional MRI cartilage assessments for OA, such as quantitative T1rho and T2 [18].

The proposed sMOAKS CDS is one example of a pseudo-continuous outcome variable that may prove useful for OA clinical trials. The CDS method allows for comparative longitudinal assessment of cartilage damage burden at all regional levels, i.e. subregional, articular surface, joint compartment, and whole knee. Because it is a scaled variable, the sMOAKS CDS may be used to compare targeted regions or combinations of regions other than articular surfaces or joint compartments. Often, the degree of severity of OA within the knee is realized at the region with the worst cartilage damage and associated bony and synovial findings. Similar to some quantitative imaging methods, assessment of scaled CDS at targeted regions or for the worst articular surface or joint compartment may be a more meaningful assessment of knee OA than at the whole joint level, where averaging across less severely affected areas may “drown out” the regional severity of disease that most contributes to patient symptoms or disease progression [25]. Indeed, it is a known criticism of summing cartilage scores that while it may offer some statistical advantages as a more continuous variable, there is loss of information as scores are consolidated across different cartilage loss levels (shallow, deep, and full thickness) and across different subregions [14]. As such, while sMOAKS may provide an advantage to the traditional analysis methods as well as the summed approach of CDS, we have shown substantial agreement regardless of the analysis methods. The choice of analyses used with sMOAKS data, however, should be tailored to the needs of the study.

In this study, we used the 16 subregion definitions described by Xie et al. [17] rather the 14 divisions of MOAKS [8]; the sMOAKS method is independent of how subregions are defined. We chose this method because the subregions for each bone are determined using landmarks entirely within the same bone, thus avoiding potential issues with joint flexion, malalignment from ligament laxity, and prior meniscal surgery. Additionally, the subregions for cartilage assessment are limited to the articular surfaces/subchondral bone. Division of the patella and trochlea into three subregions each (medial, central, and lateral) as originally proposed by Eckstein, et al. [19] can identify cartilage loss isolated to the patellar apex (also called the crista or median ridge) or the central trochlear groove, which are more common, and patella lesion locations may have treatment implications [26].

This study has several limitations. The relatively low severity of knee OA in our cohort made it more difficult to extract statistically significant agreement data at the subregion level because of the decreased frequency of cartilage defects in each subregion (supplementary material). As with most other reader agreement studies for knee MRI semiquantitative scores, interpretations of only two readers were compared. Inclusion of more readers from multiple sites would allow greater generalizability of the results. Finally, distinguishing cartilage loss at the margins of the articular surface from bony variation proved difficult, particularly in the setting of previous bony injury such as in this cohort. In patients with ACL tears, bony injury occurs with the pivot shift osseous impaction injury at the posterior lateral tibial plateau and central lateral femoral condyle, two of the subregions for which agreement values at the cartilage margins were the lowest in this study (Supplementary Material Table 1). As expected, agreement was greater for larger areas of cartilage loss than for smaller, more equivocal areas of cartilage damage involving <10 % of the subregion, commonly isolated along these bone margins of subregions where osteophytes and variations in the bone contours may confound interpretation. Likewise, small areas of cartilage damage along the borders between subregions are often difficult to place consistently in the same subregion despite being evaluated similarly otherwise.

In conclusion, we introduced a supplementation to the MOAKS cartilage subscore, sMOAKS, that adds evaluation of deep cartilage loss to differentiate less severe superficial partial thickness defects while maintaining data collection from the 2 cartilage damage levels obtained in traditional MOAKS. The new “sMOAKS” cartilage score demonstrated substantial reader agreement. Using several outcome analyses, including traditional measures and the more granular scaled sMOAKS CDS, we showed the potential utility of sMOAKS for distinguishing severity of cartilage loss throughout the joint. Further studies are needed to determine the utility of the score for longitudinal OA studies and for clinical trials using knee MRI, and define which patient cohorts benefit from the sMOAKS approach to semi-quantitative MRI evaluation.

## Editor Disclosure

The Publisher regrets that the transparency statement around the peer review process was not noted in this article: The peer review process did not involve Editorial Board Member Xiaojuan Li, and the editorial decision-making was led by editors not involved in the creation of this manuscript. The publisher would like to apologize for any inconvenience caused.

## Declaration of competing interest

The authors declare that they have no known competing financial interests or personal relationships that could have appeared to influence the work reported in this paper.

## Appendix Supplementary materials

Image, application 1
