# Supplementary material for: Capturing deep partial-thickness cartilage loss with semiquantitative scoring: A supplemented MRI Osteoarthritis Knee Score (sMOAKS)
Source: Osteoarthr Imaging. 2025 Jun 7;5(3):100272. doi: 10.1016/j.ostima.2025.100272 (PMC12372977; doi:10.1016/j.ostima.2025.100272)
Supplement: Supplementary file 1 [file mmc1.docx]

**SUPPLEMENTARY MATERIAL**

**Supplementary Fig. 1.** **Subregion division** (reprinted with permission from Xie et al.^13^). (**a**) Patella: points A and B are medial and lateral bone margins, and C is the apex. The central patella (cP) includes one-third of the A-C distance and one-third of the C-B distance. (**b**) Trochlea: points A and B are medial and lateral bone margins, and C is the posterior-most point of the trochlear groove. The central trochlea (cTrF) includes one-third of the A-C distance and one-third of the C-B distance. (**c**) Tibia: similar to MOAKS, each tibial plateau is divided into thirds and the subspinous (SS) region is defined by the tibial spines (not shown). (**d**) Femur: points A and B are defined as the intersection of a projection of the intercondylar roof (Blumensaat’s line) and the femoral margins. Point C is the midpoint of A-B. Central and posterior femoral condyles are divided by a line orthogonal to A-B at point C. lP, lateral patella; mP, medial patella; lTrF, lateral trochlea; mTrF, medial trochlea; aLT, anterolateral tibia; cLT, central lateral tibia; pLT, posterior lateral tibia; aMT, anterior medial tibia; cMT, central medial tibia; pMT, posterior medial tibia; TrF, trochlea; cMF, central medial femoral condyle; pMF, posterior medial femoral condyle.

**a**
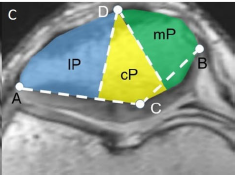
 **b**
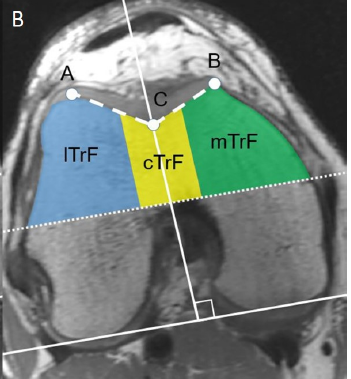


**c**
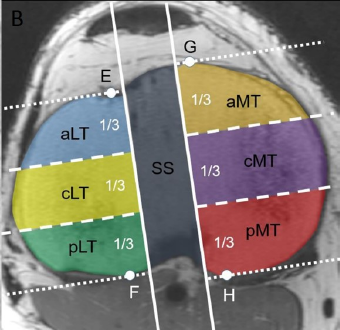
 **d**
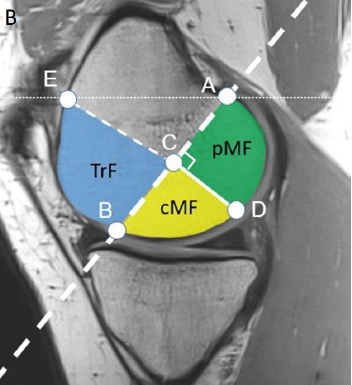


**Supplementary Table 1. Weighted kappa for reader agreement for scoring subregion deep cartilage loss size grade, taking maximum grade within an articular surface** [95% bootstrap confidence interval].

| Subregion | Inter-reader agreement | Intra-reader agreement |
| --- | --- | --- |
| Medial femoral condyle (n = 40) | 0.86 | 0.81 |
| Medial tibial plateau (n = 40) | 0.85 | 1.0 |
| Lateral femoral condyle (n = 40) | 0.58 | 0.78 |
| Lateral tibial plateau (n = 40) | 0.58 | 0.72 |
| Patella (n = 40) | 0.52 | 0.82 |
| Trochlea (n = 40) | 0.85 | 0.83 |
| Pooled over surfaces (N = 240) | 0.73 [0.58, 0.80] | 0.82 [0.73, 0.87] |

**Supplementary Table 2. Weighted kappa for reader agreement for scoring subregion deep cartilage loss size grade, taking maximum grade within a joint compartment** [95% bootstrap confidence interval].

| Subregion | Inter-reader agreement | Intra-reader agreement |
| --- | --- | --- |
| Medial femoral condyle + medial tibial plateau (n = 40) | 0.86 | 0.88 |
| Lateral femoral condyle + lateral tibial plateau (n = 40) | 0.67 | 0.74 |
| Patella + trochlea (n = 40) | 0.72 | 0.82 |
| Pooled over compartments (N = 120) | 0.75 [0.59, 0.85] | 0.83 [0.73, 0.88] |
